# Supplementary material for: Reduction of Osteoclastic Differentiation of Raw 264.7 Cells by EMF Exposure through TRPV4 and p-CREB Pathway
Source: Int J Mol Sci. 2023 Feb 4;24(4):3058. doi: 10.3390/ijms24043058 (PMC9959640; doi:10.3390/ijms24043058)
Supplement: Supplementary file 1 [file ijms-24-03058-s001.zip › ijms-2155054-supplementary.pdf]

<Supplementary figure>

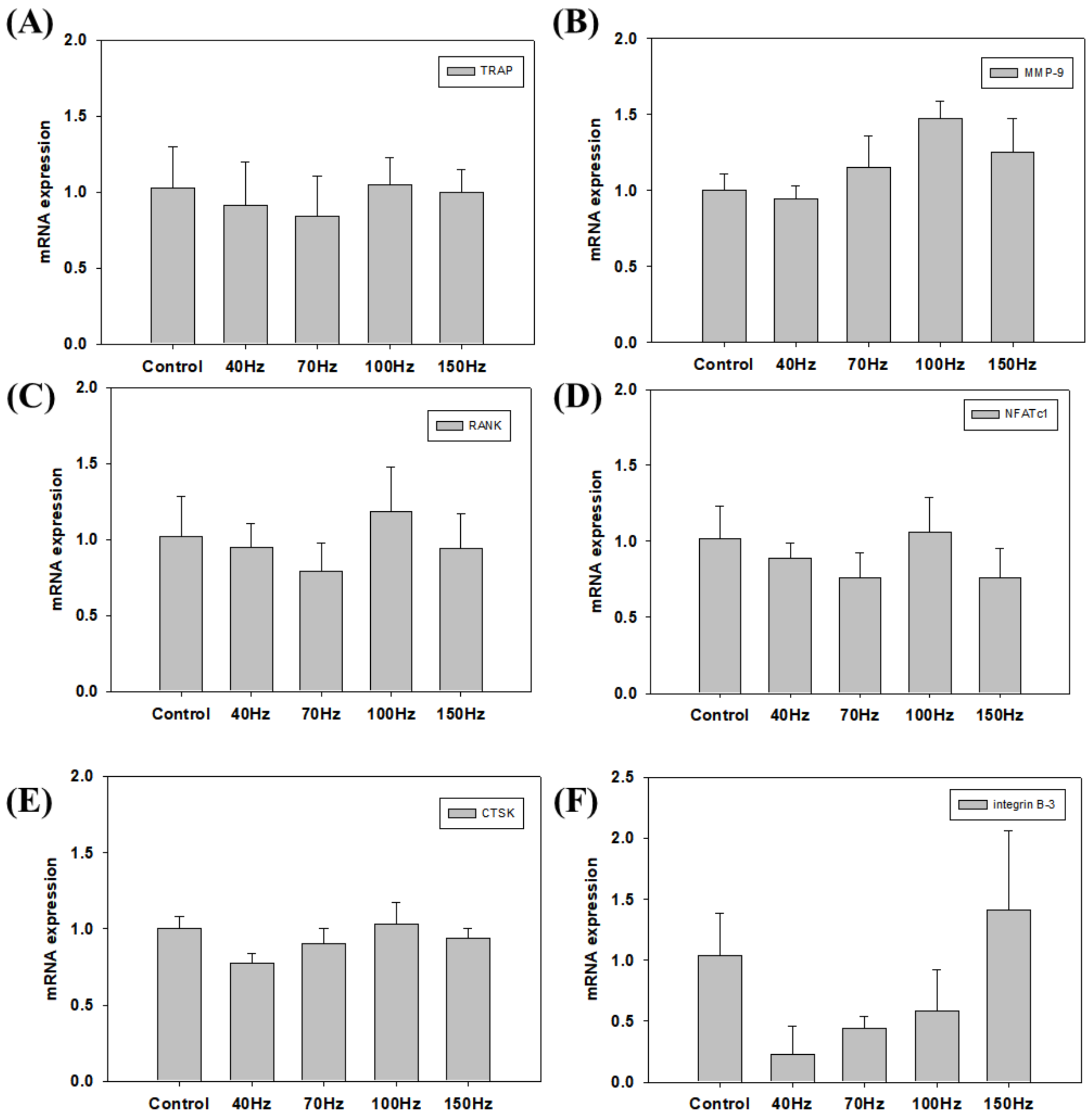

Figure S1. The mRNA expression levels of osteoclastogenic markers by RT-qPCR in Raw 264.7 cells.

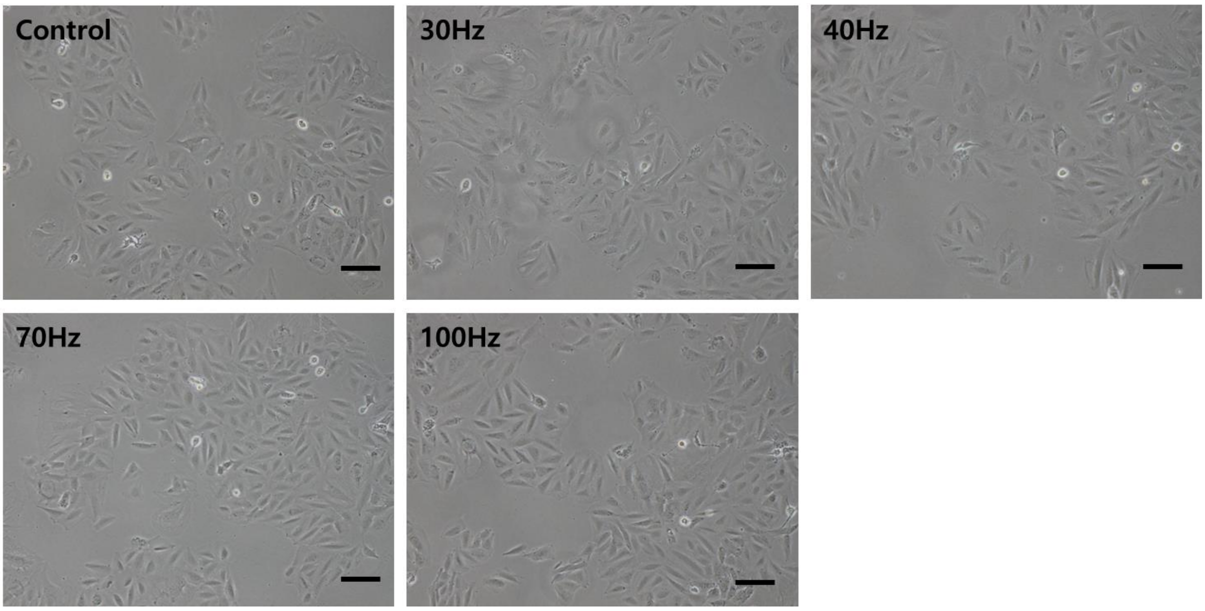

Figure S2. The morphologies of untreated (control) and various frequencies EMF-exposed SaOS2 cells after 7days.

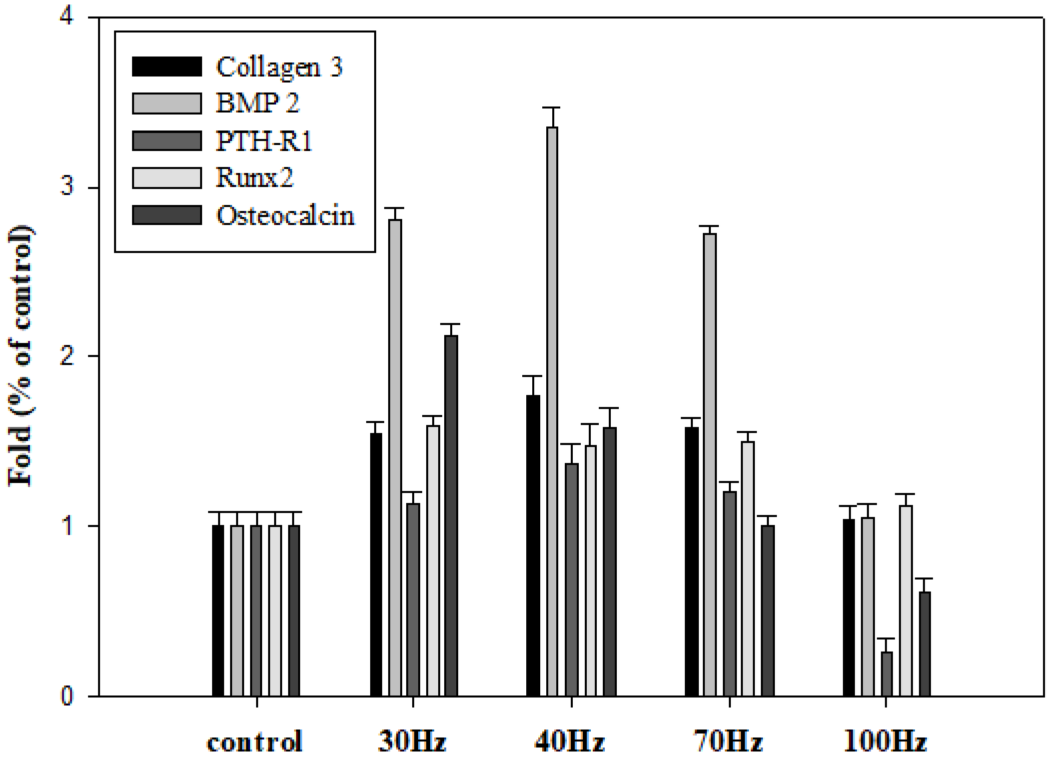

Figure S3. The mRNA expression levels of osteogenic markers by RT-qPCR in SaOS2 cells.

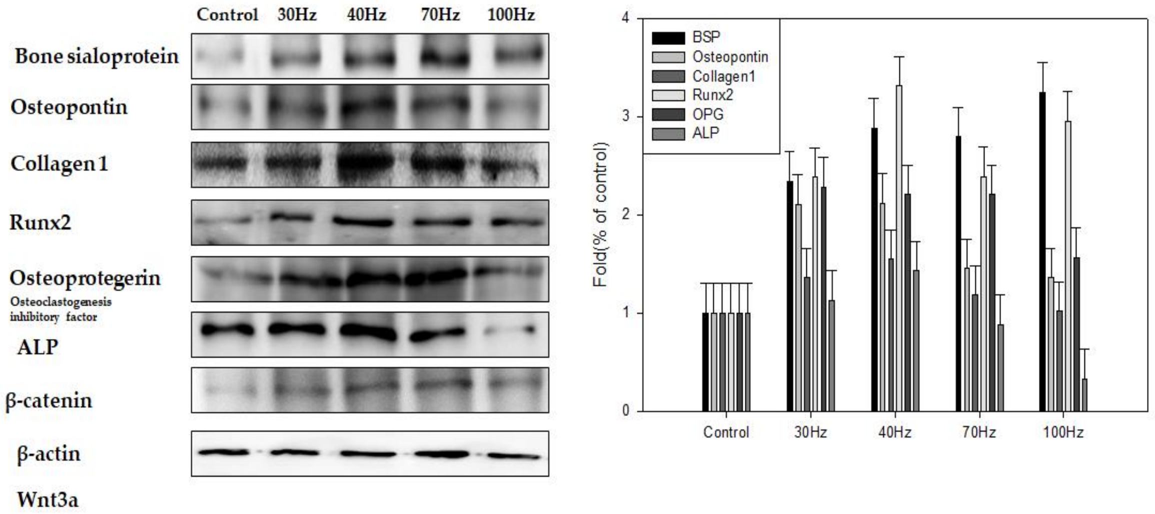

Figure S4. The expression of osteogenesis-related proteins by western blot analysis of SaOS-2 cells after culture for 7 days.

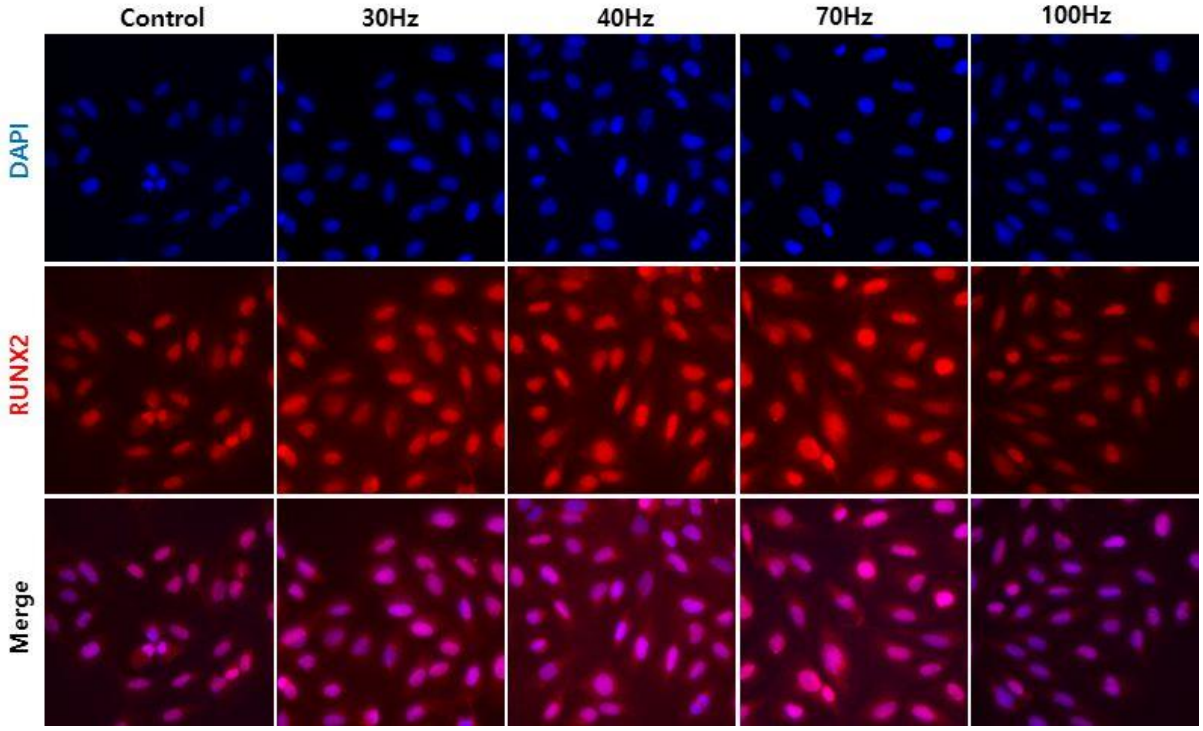

Figure S5. Immunofluorescence staining of Runx2 after various frequencies EMF-exposed SaOS2 cells.
